# Practice of Medicine

**Published:** 1825-04-01

**Authors:** 


### VIII.

1. *A Compendium of Theoretical and Practical Medicine; comprising, with the Symptoms, Diagnosis, Prognosis, and Treatment of Diseases, a General Review of Physiology and Pathology, together with an Estimate of the present State of Medical Science.* By David Uwins, M.D. Licentiate of the Royal College of Physicians in London, and late President of the London Medical Society, &c. Small octavo, pp. 386. London, January, 1825. Longman & Co.

2. *A Nosological Practice of Physic, embracing Physiology.* By George Pearson Dawson, M.D. Octavo, pp. 380. London. 1824.

Dr. Uwins seems to anticipate a very natural question on the appearance of a new compendium, namely:—

"With the volumes of Good and Gregory, Temple and Thomas, in the hands of our students, what can be wished for more? and if a compendium be required, is it not furnished by the Vade Mecum of Dr. Hooper?" *Preface.*

When people ask themselves questions, they seldom find much difficulty in answering them. After eulogizing the volumes abovementioned; all and each of which are pronounced to be excellent in their way, a hiatus is discovered, and a field for industry and genius proclaimed.

"But still a *compendious combination of pathology with practice*; of doctrine with fact; of present-day science with former information, has long appeared to me a decided desideratum in medical literature. Under this impression, the items composing the present tract have been put together; and, under this impression, the tract itself is, at length, after many unlooked for delays, respectfully presented to the medical student." viii.

By the author of the second work on the above list, we are informed that the production now offered to the public is the result of 28 years dedicated to the study and practice of medicine.

"The object which the author had in contemplation was to exhibit general views of the principles and treatment of diseases, interspersed with classical, physiological, and pathological facts and observations. Dissatisfied with existing nosologies, he has constructed one for himself, which is simple, intelligible, and consentaneous with his own experience." *Advertisement.*

The plan and execution of these two works differ materially, one from the other. Dr. Dawson is always fixed in his opinions, positive in his assertions, and somewhat dogmatical in his didactics—Dr. Uwins is constantly trimming (we mean in the first part of his work, "*General Physiology and Pathology*") between opposing doctrines—and, like the philosopher in Rasselas, always concludes without any thing being concluded. The language of Dr. Dawson is always good—often elegant. That of Dr. Uwins is the most tortuous, rugged, and *designedly* uncouth, which we remember to have seen in any modern author. We say *designedly;* for no man ever took such pains to disguise himself and distress the ears of his readers, as Dr. Uwins. We tell him this plain and uncourtly truth, because we respect him, and are sorry to see him injure himself through mistaken notions about the advantages of a singularity of style.

Dr. Uwins's work is divided into three parts—the first embracing "General Physiology and Pathology"—the second, "Synopsis Nosologiæ Methodicæ"—the third, "the Symptoms, Causes, and Treatment of Diseases." The first part is his own—the second is Cullen's—and the third is public property, the child of adoption by hundreds before Dr. Uwins.

Dr. Dawson has constructed a scheme of nosology for himself, on which he has engrafted a short system of pathology and practice, apparently the result of his own observation and reflection. Dr. Dawson has, in our humble opinion, taken a very judicious view of the causes, pathology, and treatment of diseases; and although he is, as we said before, sometimes a little too positive and dogmatical, and generally too concise; yet we have seen few works of this description, in which there are so many proofs of sound judgment, as in this unostentatious performance.

It is with the first part of Dr. Uwins's work, of course, that we can have any thing to do. It opens with the often discussed question—"*is medicine a science of certainty or conjecture?*

—"*What claim has it upon public estimation?*" In respect to the first question or part of the question, every body knows that medicine contains a little certainty, and much uncertainty. Of *conjecture*, there has been no lack, from the earliest ages down to the present time. The second question, respecting public estimation, is answered by the public itself. There is scarcely a human being, gentle or simple, pious or profane, from George IV. to the most wretched inhabitant of St. Giles's, who will not fly to medicine the moment that disease assails. *In health*, the saint will tell you that this life is a winter's day of sorrow, and that the things of this world are beneath the notice of a man of wisdom. But let him be overtaken by disease, and he will drain every phial of physic with as much eagerness as the mere man of this world. *In health*, the bon vivant, the debauchee, the scoffer, and the sceptic, will all laugh at physic, and quiz the doctor. But place these worthies on the bed of sickness, and it will soon be—" Oh! dear doctor, how I did long for your arrival!" With these facts hourly before our eyes, where is the necessity for agitating the question of "public estimation." The doctor does not *force* his services on the public, or, like the lawyer, enveigle the client into an expensive suit. His attendance is perfectly optional on the part of the rich—*and generally gratuitous when poverty makes its appeal.* Do we see much of *this last* at the bar, or even in the pulpit, where it might be expected? No! But the public see and feel these things—and estimate accordingly.

Nosology, by Dr. Uwins, is voted an absurdity.

"The very nature, then, of medical evidence is totally inconsistent with attempts to identify and arrange diseases, as you name and classify a plant by its generic and specific characters; and the assumptions of the nosologist are altogether based upon an error in principle. They suppose the existence of something which really does not exist, and it is amusing, or rather perhaps I should say humiliating, to open volumes, and find learned authors elaborately discussing the legitimate ranks and seats of morbid affections in nosological schemes, when the scheme itself, in respect of its abstract nature, is as little entitled to regard, as the question of the schoolmen on the number of angels that might dance upon the point of a needle without interfering with each other's evolutions—angelic being assumed subtile substance, as opposed to the materiality of the needle's point." 5.

After this angelic dance upon needle points, we certainly were a little astonished to find Dr. Uwins copying Cullen's Nosology from alpha to omega, as a very useful portion (50 pages) of his "COMPENDIUM." Dr. Uwins could not help anticipating this feeling on the part of his reader, and has apologized accordingly.

"With these announcements of feelings in reference to nosology, my reader may deem me inconsistent in selecting *any* scheme for the present purpose of producing an epitome of medical science. But the fact is, we have only a choice of evils, and must endeavour, out of these, to select the one that makes the nearest approach to good." 6.

The nearest approach to GOOD would apparently have been the nosology of GOOD himself—but our author thought otherwise, and therefore selected the least of two *evils*.

The question respecting nosology is, we imagine, very much narrowed, by all men of observation and reflection. The order and arrangement of nosology facilitates the acquisition of our *elementary* knowledge of diseases—it has little effect on our *practice*, after experience has been acquired at the bedside.

*The Circulating Function* is the first physiological subject discussed by our author. The pros. and cons. for and against the vitality of the blood, are delineated, and then, as usual, a middle course is steered—that is, Dr. Uwins supposes the blood to be vital in one sense, and not vital in another. "To call this fluid the vital fluid, is, in one sense correct, in another erroneous."

"As the primordium of all other organic parts, solid or fluid, the blood has some legitimacy of claim to the epithet distinctly of vital; but I would be bold to say, that in Mr. John Hunter's application of the term it has not. All parts of an organized being are properly vital, while they constitute portions of the bodies integral, and to fix, even by implication, vitality (which is but an expression of the whole), upon certain parts, is to fall into the error of the *entity* philosophers, to search for things that are not to be found, and which have no real existence." 8.

In respect to the mechanical part of the circulation, our author trims his "neat-built wherry," between those who advocate the doctrine of alternate dilatation and contraction in the arteries corresponding with the systole and diastole of the heart, and the other party who deny that the arteries contract and dilate according to the filling and emptying of the left ventricle of the heart.

"With respect to the controversy to which I have just adverted, and aimed at giving an outline, I think *a sort of intermediate opinion* would in this, as in most controverted points, prove nearest the truth. Certain it is that the capillary arteries possess a projectile power of considerable independence upon the heart; but it must be recollected, that in these vessels, or rather in this part of the arterial organization, the blood moves rather in a continuous stream, than in the jerky manner of the larger branches; this independence, then, of the capillary circulation upon the heart, together with the altered manner of movement at the point where such independence commences, might be taken rather in proof of, than an argument against the doctrine of arterial action; but still we find in both sides of the question, considering it as an abstract one, considerable difficulties; and the great problem respecting the actual *quo modo* of the blood's transmission from, and reception into, the source and centre of circulation, remains yet unsolved." 19.

Inflammation, as one of the deviations from health in the circulating system, is, of course, descanted on, and the two great contending hypotheses—*diminution and increase of action in the capillaries*—are examined with our author's peculiar logical acumen. Dr. Uwins is happy here, as elsewhere, in finding the golden mean.

> *Auream* quisquis *mediocritatem*
> Diligit, *tutus* caret obsoleti
> Sordibus tecti—caret invidenda,
> Sobrius aula.

"If there be any correctness in what has been above advanced, in reference to the essentials of inflammation, it would seem, moreover, to follow, that the dispute is somewhat idle and unmeaning, whether inflammation be weakness or strength, action or torpor, obstruction or other impediment: it is all and every thing as the general frame and local incidents shall vary; and, both as to pathological essence and practical indication, abstract views ought to be kept as much as possible from influencing judgment. We shall afterwards have occasion to see that stimuli are called for under some circumstances, and anti-excitants under others; the disorder being nominally the same, nay, that both principles may be brought correctly and beneficially to bear upon one and the same condition." 26.

From inflammation we ascend to fever, and here it will be seen that Dr. Uwins has judiciously steered wide of the errors into which Clutterbuck, Broussais, Marcus, Mills, and their disciples have run. The extract is rather long; but it furnishes a good sample of the manner, as well as the matter of our author.

"But is fever, the question still recurs, a local or general disorder? Does it happen as a consequence of some primary irritation falling upon a *part* of the body, and in this way producing a series of associate actions differing from healthy movements; or, is the first impulse made upon the totality, as it were, of the frame, the perturbed movements being nature's hurried efforts to set all to rights again, *as the surprized soldiers of a suddenly beseiged town would hurry some to the citadel, some to the outposts, and some they know not where, but all intent to repel the attacks of the enemy.*

"It has always appeared to me, that those theorists who will have a local origin of fever, as lords to rule over them, overlook too much what is of every day and ordinary occurrence in the way of febrile induction, without the perturbation to which I allude becoming so forcible or permanent as to engender positive or specific fever. If I am subjected to a certain quantum of fatigue, or if things about me become hurried and irregular instead of due and orderly, I become feverish, my skin is hot and dry, my spirits depressed, and my thoughts and perceptions confused. Now, another individual, not perhaps more robust than myself, shall be exposed to the same source of derangement, but shall either not at all be affected, or shall be affected in a different manner—what would break down his energies, would lock up mine—what would exhaust him, would confuse me. Withdraw the morbific excitants in either case, and my restoration shall at least be as speedy as that of my friend. Have, then, my brain or mucous membranes been *inflamed* under this condition of things, or has any local mischief been brewing, which shall thus have been speedily dissipated? Is not the more consistent explanation of the affair this, that the whole machine has *at once* fallen out of its consentaneous order, and demands to be re-adjusted before actual health can be predicated? And the same principle would appear to apply, and the same process but in a more marked manner, to take place when the exciting causes act with more intensity, so as to produce real and lasting, in the place of ephemeral and slight disease. Does inflammation never take place in fever? Most certainly it does, and that very frequently too: but the inflammation, whether of the brain, or the membranes, or viscera, is not primary and characteristic, but secondary and incidental. The identifying, then, of fever and inflammation is an instance of modern generalization which does not appear to me to be countenanced by fact. I shall be asked what is the difference between pneumonia or inflammation of the lungs, and fever, and I must confess the question presents some difficulties, in reference to febrile pathology altogether: since, if we say that inflammation of the lungs is a local, while fever is a general disorder, it may be replied, that a general commotion, marked by shivering and shrinking, often ushers in the one as well as the other. The truth is, I believe, as I shall afterwards have occasion more particularly to point out and illustrate, that the idea of local disease altogether has been too loosely admitted, and that topical is, for the most part, a mere manifestation of universal affection. Still there is a distinction between fever and inflammation. Who has not observed, for instance, how different inflammation of the lungs and its attendant fever is from fever and its attendant inflammation of the lungs? In the latter case, we have the *languor cum arteriarum pulsu inordinato* of authors, the peculiar animal depressions that characterise genuine fever; while, in the other case, there shall be the height of topical pain and systematic disturbance, without this attendant expression of sentiment, or cerebral disorder. "Cerebral disorder," would one class of systematics repeat after me, you have now conceded to us all that we desire; we contend that inflammation of the brain, and fever, are one and the same thing, and you, in terms, have subscribed to our postulate.

Not so. Brain disturbance is very different from brain inflammation; and although the febrile state very frequently indeed becomes a real inflammation of the encephalon, it does so merely from the circumstance of the sensorial organ being the organ most implicated with the deranging influence, and thence most vulnerable to the attacks of veritable inflammation.* In a word, and to conclude, that species of deviation from the healthy standard to which nosologists have applied the term fever, is a consequence of an hitherto undefined, or at least unexplained, derangement of the nervous organization, manifested by sensorial disturbance and vascular irregularities, to which, for want of more precise notions and definite conceptions, the words spasm, and inflammation, and congestion, have been applied, with some degree of rectitude, it must be admitted, but still with an objectionable freedom and decision, inasmuch as, after all, even allowing the legitimacy of the terms, they are but statements of results and incidents, while they lay claim to developments of source and essence." 38.

Our readers are well aware, that the essential points in the foregoing extract have been urged and upheld in this Journal for more than ten years past. We are happy in having the support of Dr. Uwins on this occasion: but we expect to be called upon soon for a more elaborate defence of our opinions—and from this task we shall not flinch.

*Contagion*. The discussion of this important question naturally arises out of the subject of fever—and here our author "ut mos est," closely adheres to the advice given by Phœbus to Phaeton—"in medio tutissimus ibis." The following passage is certainly in Dr. Uwins's best style, and as such we are happy to introduce it to our readers."

"What are the kinds and species of fever? Some will tell you fevers, whatever types they assume, are one and the same thing; and that there is no difference, but in the imagination of the nosologist, between the virulent plague of the Levant, and the low fever of a London alley. Others, again, divide and subdivide to an enormous amount, under the feeling that every new modification of the ailment, in acknowledging a distinct source, demands a distinct appellation. Here, as in almost all disputed points, we, perhaps, shall find truth taking its stand at about mid-

"* Were fever inflammation of the brain, it could not subside, as it often does, in a moment almost of time, after a long continuance, without leaving any traces behind it of organic mischief. Had it been inflammation that was going on during the long protraction of continued fever, the common and necessary consequences of inflammation could not fail of invariable manifestation. Fever, it is readily allowed, does very often, indeed, induce topical and chronic ailments, and, in these cases, a species of inflammation has grown out of the original disorder; but events of this kind are not uniform, and the topical sequelæ are not always in the head."

distance between the poles of contention. It is the opinion of some, that genuine fever never can originate without the agency of what is called contagion; others, on the contrary, as I have before intimated, go so far as to deny the existence of contagion altogether, except among a few specific distempers; while a third party are ready to admit contagion as an agent in the production of fever, but, at the same time, contend that the disorder may be elicited from other powers, such as the undue operation of cold and heat, famine, repletion, filth, and mental agitation.

"What is the meaning of the word contagion? It is, by the general acceptation of the term, a something in which, coming in contact, either immediately or intermediately with the body of an individual previously in health, engenders a disease in that individual of a nature precisely similar to its own—it implies, in a word, specific poison. What is infection as opposed to contagion? If I take a journey to Constantinople while the plague is raging in that city, and if, by consequence of my residence there, I fall sick of plague without having come in contact in any way with the matter that shall have emanated from the body of another person who is the subject of plague, I receive an infectious malady. Here, then, is the difference between an infection and a contagion—that the one is atmospherical, or only through the medium of the unhealthy air communicable, while the other implies communication, either direct or indirect, with a something issuing from the body of the sick. In either case, indeed, a contact with a poison is, strictly speaking, supposed; and hence, there is some impropriety in basing the notion of difference upon the assumption of different operation. I am disposed to think, indeed, that much of the intricacy that attaches itself to the question of contagion and infection, originates in this very source; that is, a variety in *modus operandi* is presumed, which is really nonexistent. Let us imagine that a patient dies in the Levant, of the disease called plague, and that the clothes in which he died are conveyed to this country, embued with the poison of plague. An inhabitant of our metropolis procuring and putting on these clothes, might be affected with a certain dose, if I may so say, of disease; but still, though the virus might be as active *in se* as, had the same appropriation been made of the habiliments at another quarter of the globe, yet the resulting distemper might nevertheless, be various—and in that case, you would say of the individual that he had taken an infectious, as well as a contagious distemper. In this way, then, the question may be viewed as standing out in a tangible form. Is there any reason to believe that some disorders are entirely of an abstract nature, dependent solely upon their several exciting sources, without reference to time, place, or circumstance? or does the modifying power of place always, to some extent, operate upon the nature and aspect of a distemper? For my own part, I am disposed to think it does, so that, although in one sense I incline towards the sentiments of the anti-contagionist, I, in another, recede from his inferences, even much further, than does the contagionist himself; for I believe, that from the most decided case of small-pox, down to a nursery cold, there is something communicable in the maladies, and something operative in the atmosphere." 40.

Passing over the exanthemata, the profluvia, and other heads, we come to the "sensient and motive function;" but we are too sick of the theories of "nervous agency" to enter upon this subject here. Dr. Uwins takes a desultory review of the different hypotheses regarding the nervous system, and concludes without any thing being concluded.

The "ASSIMILATING FUNCTION" next succeeds, and a series of remarks on the physiology and pathology of this portion of our system, is given by our author. We do not see any thing, however, which particularly requires comment here.

We have now surveyed the first part of the work, and have presented sufficient specimens to enable our readers to form a conception of Dr. Uwins's manner and execution. To the *matter* we have little or no objection to make, as Dr. Uwins has steered, in general, a middle and safe course, between contending theories. But, to our author's style, we decidedly object. It is exceedingly inflated, in many places, and not seldom disfigured by unnatural metaphors. We refer the reader, for an example, to our author's illustration of the onset of fever, in which he compares the human frame to a suddenly-beseiged town, where all is confusion and tumult. Surely such metaphors as these are not calculated to make a favourable impression on his scientific brethren. To us it appears that Dr. Uwins has completely ruined his professional style and language by his long habits of writing on medical subjects for the popular magazines and reviews. In this species of composition, precision is exchanged for point—perspicuity for brilliancy—homely and *appropriate* illustration for gaudy and *irrelevant* metaphor—and last, not least, sober argument for seductive rhetoric. These observations are well exemplified and confirmed in the case of Dr. Uwins's celebrated predecessor in this walk of literature—the late Dr. Reid. That physician had great command of language, and he pushed his power to the utmost; but what was the consequence? His whole work is a tissue of fine words, polished sentences, and sparkling metaphors, conveying either no ideas at all, or those of the most common place, tinselled, lacquered, and gilded for popular exhibition. In short, Dr. Reid's *brilliant* pictures of *Melancholy* constantly remind us of chimney-sweepers on a May-day—all gold, in appearance, outside—and all soot, in reality, within.

Dr. Uwins has run into an extreme the reverse of Dr. Reid's. Instead of selecting his expressions and polishing his sentences, he appears almost to have studied uncouthness—and in pursuit of colloquial simplicity, he has not seldom bordered on vulgarity. Of this he seems a little conscious himself, as may be gathered from the following passage in his preface.

"With respect to its construction, I have aimed at a familiarity of style. In this aim I have probably failed, and may be found to be dogmatic and vulgar, while wishing to be impressive and colloquial; if so, I have failed in power rather than in feeling, and must be pardoned on the score of good intention. To one thing I can conscientiously and confidently speak; namely, sincerity of purpose; and the circumstance may, in part, be taken as a guarantee for that sincerity, that some of those individuals, with whose doctrines the greatest freedom has been used, are among my most respected friends." viii.

For all these professions we give our author full credit—for not one of his friends entertains a higher respect and esteem for Dr. Uwins, as a gentleman and physician, than ourselves. Our strictures are purely critical of the book, and have no other reference to the author.

We now proceed to the main part of the work, the symptoms, treatment, &c. of diseases, passing over, of course, the nosology of Cullen. This part, then, appears to us (though by far too concise) a fair epitome of the best established matter that could be drawn from standard sources of information in the English language, mixed with a certain portion of personal observation, of no great extent, and followed by formulæ for the exhibition of the remedial agents recommended. We do not think we could better or more fairly characterise this portion of the volume, if we were to employ whole pages for the purpose; we shall, therefore, offer a single specimen taken without the least selection.

"Genus XIV.—Anxiety; Gastritis.

"Typhoid pyrexia; heat and pain in the epigastrium, increased by taking any thing into the stomach; an inclination to vomit; and immediate rejection of the ingesta; hiccup.

"*Symptoms.*—Gastric inflammation brings with it extreme depression of spirits, and prostration of strength; the pulse is often very small, at the same time that it is rapid and contracted. Urgent thirst prevails, and the pain is sometimes increased by pressure upon the region of the stomach. The tendency of the disease is often towards gangrene, unless resolution be early accomplished; and if gangrene occur, although the heat and pain remit, the pulse increases in rapidity, hiccup and coldness of the extremities succeed, and life is soon terminated.

"*Remarks.*—Gastric inflammation of an acute and phlegmonous kind, is of much less frequent occurrence than might *à priori* be supposed.

It has been observed, that in collections of morbid preparations, there are fewer instances of disorganization from this cause, than from almost any other; the diseased appearances of the stomach that are met with being, for the most part, either those of slow and chronic inflammation, or irritation of a specific nature, as of schirrus, affecting either the pyloric or cardiac orifice of the organ.

"*Causes.*—Acrid or indigestible substances taken into the stomach; alternations of temperature; draught of cold water, while the body is hot; suddenly repelled eruptions, and metastasis, especially of gout.

"*Diagnosis.*—From spasm by the pyrexial symptoms, and burning heat; from enteritis by the seat of the pain; also by the burning sensation, vomiting, and tendency to hiccup, even before the gangrenous termination, which in enteritis is a mark of gangrene having commenced.

"*Prognosis.*—If, in the course of two or three days, the pain and sickness cease, while the pulse becomes more free or full, and diminishes in frequency, the urine depositing a sediment, and the bowels becoming spontaneously loose, a favourable termination may be expected. The unfavourable symptoms are a continuance of disease for several days, the pulse increasing in frequency, and hiccup remaining in spite of the medicinal processes.

"*Treatment.*—General blood-lettings, and the free application of leeches to the epigastric region; afterwards warm fomentations, and a blister, the copious employment of mucilaginous diluents, such as linseed tea, good barley water, with gum acacia dissolved in it. Emollient anodyne clysters are likewise to be injected.

℞ Tincturæ opii f ʒ ss.

Mucilaginis amyli.

Aquæ puræ āā f ℥vi.

Fiat enema.

Or these may be more purgative.

℞ Olei ricini f ℥iij.

Decocti hordei f ℥v.

Fiat enema.

"Endeavour to allay irritation by the exhibition of the following draught.

℞ Potassæ subcarbonatis ℈j.

Succi limonis f ʒ ss.

Tincturæ opii ♏ vj.

Aquæ puræ f ʒ ss.

Fiat haustus.

"Pills, with a grain of calomel and a grain of opium, administered three or four times in the course of the day, will be found sometimes to allay pain, and arrest inflammatory action.

"The erythematic inflammation which is often consequent upon a long habit of intemperance, especially in the use of spirituous liquors, is, for the most part, best met in the way of medicine, by very small doses of mercurials joined with one or more of the vegetable narcotics.

℞ Pilulæ Hydrargyri.
Extracti Hyoscyami
——— Conii āā gr. ij.
"Fiant pilulæ duæ, nocte maneque sumendæ.

"When the irritation is of that nature which leads to schirrus of the pylorus or the cardia, the above combination of medicinals will be found useful in allaying pains, and thus mitigating the sufferings of the patient." 188.

The principal defect in the work, is the great neglect of pathology—that is, an account of the changes which parts undergo during disease, and the *post mortem* appearances. If, in the next edition of the work, Dr. Uwins would take the trouble of adding a head of this kind to each genus of disease, he might cancel the whole of the first division of the book, to make room for this additional specific information, and thus render the performance a truly valuable acquisition to the student and junior practitioner.

We now come to the second work on our list, though the first in order of publication. Dr. Dawson has arranged his nosology into five orders, viz:—1. Febrile diseases—2. Inflammatory diseases—3. Nervous diseases—4. Cachectic diseases—5. Functional diseases. This nosology may be "simple, intelligible, and consentaneous with his own experience;" but we fear it will be found as imperfect as other systems of nosology now in use. This we do not impute to our author as a fault—because we are convinced that any thing like perfection in nosology, is unattainable by man.

Although pretensions are not made to much originality (strictly speaking) in the matter of Dr. Dawson's volume, (which indeed it would be unreasonable to expect) yet we almost always find him judicious in the selection of materials from others—and correct in the judgment which he comes to as the result of his own observation and reflection. These two qualifications are the most important ones which the author of a systematic work can possess. To exhibit much novelty in a performance of this kind, would be to exhibit much error. The development of new and *true* knowledge is extremely slow—and must come piece-meal from a number of labourers in the vineyard of science. No man ever did or ever will proclaim a *new system* of medicine, of more than a very temporary duration. Brunonianism is a recent and memorable example in our own times. But much may be done by systematic writers, in correcting a few of the errors of their predecessors, and adding a little to the store of established facts. This is all that can be rationally looked for, in the present state of things.

Dr. Dawson has avoided the defect which we noticed in Dr. Uwins's work, namely the omission of the pathological state, as ascertained by dissection. Dr. D. has appended to each disease a short sketch of the appearances *post mortem*—a thing too much neglected in all our English systems, but which is now indispensable.

The classical allusions in this work are pleasing to the reader, and creditable to the author. They are generally appropriate; but, we think, sometimes a little overstrained, if not erroneous. Thus, when on the subject of dysentery, our author introduces the following classical allusion.

"Cornelius Nepos, in his life of Atticus, the friend of Cicero, records the particulars of the disease that destroyed him, which was most probably a dysentery. He observes, that he had been remarkably healthy for thirty years, when he contracted a tenesmus, which was deemed to be of no importance. Three months passed without his experiencing any pain, when "subito tanta vis morbi in unum intestinum prorupit, ut extremo tempore, *per lumbos fistula putris eruperit*: atque hoc priusquam ei accideret, postquam in dies dolores accrescere, febremque accessisse sensit." 159.

Now we confess we cannot see any positive proof of dysentery, as the cause of death, in Atticus. To us it appears much more like lumbar abscess. It has not been our lot ever to see dysentery without pain—and the "*per lumbos fistula putris eruperit*," has little to do with the disease under consideration. In fact, we cannot construe it in any other sense than *lumbar abscess*.

We can select but a few specimens of the work before us, to shew the matter and manner. Our first example shall be from the 1st class—or febrile diseases. After making some acute remarks on debility and spasm, our author proceeds thus to comment on some more recent doctrines.

"My friend Dr. Armstrong, distinguished by his successful writings, has divided typhus into simple, inflammatory, and congestive;* but these can only be viewed as different stages of the same fever, arising accidentally, frequently blended, and therefore unworthy to be considered as constituting three grand divisions. Yet it must be admitted, that Dr. Armstrong's varieties,—they are nothing more,—are of considerable practical utility, and are founded upon the different phenomena of the disease, which are of common occurrence. Unquestionably we often meet with a simple typhus, where, from beginning to ending, no inflammatory or untoward symptom occurs: in like manner, typhus sometimes shews itself with a remarkable disposition to inflammatory affections of particular organs; and, lastly, it cannot be denied, but

"* Armstrong's Practical Illustrations, &c. London, p. 9. 1816."

another kind of typhus does, although more rarely, appear, where it is neither simple nor inflammatory, but a fever in which there is a great and dangerous oppression, without any efforts in the system towards re-action. Such a fever is named congestive, which is a popular, and not a philosophical, term—and, like most popular terms, it is incorrect. Congestion, if it mean any thing, must mean obstruction—what obstruction? It is presumed, the patrons of the word mean—of the vascular system; for what else can they mean? Now congestion, in the sense it is used by the authors of it, having a general application to the whole of the vascular system, is, during life, physically impossible; for congestion of the vascular system is death, and what actually takes place in death. How can a system so important be congested, be obstructed—for that is the true import of the word? The fact is, in congestive fever,—the word is used for the sake of distinction,—there is not, and cannot be, any congestion, or obstruction of the whole of the vascular system; but the vascular and nervous systems are greatly oppressed by the violence of the morbid power, and this oppression imperiously demands venesection, which relieves the body from an injurious load, and induces re-action. Dr. Good, so laudably sensible of the faults of other nosologists, has made fever a disease of the vascular system, and Dr. Armstrong has done nearly the same; yet the nervous system, as will be adverted to hereafter, is equally, nay, perhaps, more engaged; so that, in accordance with Good's own doctrines, fever might have ranked under his *Neurotica* with great propriety. If ever any disease deserved the name of universal, that disease is fever, which acts on the whole body at once with resistless violence. No candid man, who attentively observes the phenomena of fever, can think the vascular system is principally affected; since he ought to know that the affection is universal, and particularly involves the nervous system. Dr. Clutterbuck, with an ingenuity and talent which do him honour, has asserted and attempted to prove fever to be, at all times, an inflammation of the brain.—This will not do: it is to mistake an occasional effect for a constant cause. But for this unhappy prepossession, he would have ranked high among the authors on fever; since it is not easy to discover his superior. No doubt the brain is affected in fever; but this is attributable, not to inflammation, but to the disorder of the nervous system. Inflammation sometimes attacks the brain, and sometimes any other viscus, as may be expected during a hurried circulation, where the weakest organ, from an influx of blood, becomes *bona fide* congested, which congestion is the essence of inflammation. Dr. Reid, of Dublin, refers fever to the spine,—another unhappy prepossession, retarding instead of advancing truth. Such views are truly unphilosophical: they narrow an important subject, which disdains such petty limits. If fever have "a local habitation and a name," it is in the whole body, and not in a particular organ or organs. These cerebral, spinal, and congestive attachments, strongly resemble the love-infatuations of early life, which commence in misconception, are perpetuated in folly, and clung to with an obstinacy proportionate to the worthlessness of the object." 33.

We can make room for only one more extract, which, however, is rather a long one, but one of much interest, and containing a good deal of originality.

"III. *Gastrodynia.* Excruciating pain in the stomach, of uncertain occurrence and duration. Health commonly unaffected.

"Gastrodynia, sometimes, but not always, connected with dyspeptic symptoms, is, and ever has been, a common and agonizing disorder in every age and nation, rendering the subject of it miserable, and inadequate to every exertion, whether of pleasure or business. It can scarcely be termed dangerous; for it often exists a number of years, without destroying the general health. What can be its cause it is difficult to surmise:—probably, flatus in the stomach, or an acrimonious state of the gastric juice:—in other instances, doubtless disease of the stomach; but here death speedily ensues.

"Virgil, a name well known to every scholar, "was often troubled with a pain in his head and stomach," clearly the consequences of dyspepsia; for he led a sedentary life, and was of a "sickly constitution and swarthy complexion.*" Cicero†, the most anxious of mankind, as he himself acknowledges, suffered severely; and was only relieved by vomiting a fluid which he thought was pure bile. Claudius, the fourth emperor of Rome, endured grievous pain in his stomach, so as to lead him to contemplate suicide:—"*stomachi dolore. Quo se correptum etiam de consciscenda morte cogitasse dixit.*‡" A certain well-known London secretary was miserable, from a painful feeling in his stomach, something similar to rats eating the villous coat. Gastrodynia is a more common complaint than the world imagines; yet it attracts no notice, and gains no sympathy, inasmuch as the health is unimpaired and the appetite usually good, although the digestive organs are always more or less disordered. The spirits are invariably depressed; and, during the paroxysm, every sensation is distressing, and every feeling horrible. The time of the accession of the paroxysm, and the duration of it, are alike uncertain. The pain may come on in the morning, afternoon, evening, or middle of the night:—according to my own experience, night and morning are the most common periods. With regard to the continuance of the pain, it may be an hour, two, three, five or six, and considerably longer, if permitted to proceed unmolested. A hearty meal or a copious drink of a stimulating fluid sometimes brings temporary relief. The pain is aggravated by walking, and slightly mitigated by reclining on the left side, and applying pressure by the hand. In some cases the pain is always in the stomach, and in the stomach only; but in others the pain does occasionally, for a short period, quit that organ, and, as it were, indifferently and fancifully affects the back, sides of the spine, or the integuments covering the sternum and ribs,

"* Davidson's Virgil, in Vita Virgilii, vol. i. London. 8vo. 1748."

"† See his Letters during his Exile. Valpy's Latin Edition. 12mo."

"‡ C. Suetonius Tranquillus, in Vita Claudii."

thus implying the presence of flatus in the stomach. The pain itself is of a peculiar, and even of a varying nature. It is not acute, it is not lancinating, it is not spasmodic; it is neither sickening nor dragging:—they best can tell who have felt it:—the pain is *sui generis*: it is of an excruciating aching kind, and of the most soul-depressing nature. The patient can stand upright, but he naturally bends the body, and presses with his hand, to obtain partial relief. Walking, reading, talking, or sleeping, are absolutely impossible. I have known a gentleman lie on the floor in agony, and have three distinct attacks, of three, four, and six hours, during twenty-four hours. Sometimes the stomach feels empty; at other times, it seems distended, and gives rise to bitter or saltish eructations. Yet the patient, even on the rack of pain, is not ill; and the instant the pain ceases, he is as well as he could wish. It deserves to be noticed, that this pain scarcely ever exists with pain in the head; and if, which seldom happens, the head becomes affected, an almost instantaneous abatement of the gastrodynia occurs. According to my experience, gastrodynia is comparatively harmless, when neither vomiting nor emaciation prevails.

"Such is a faithful history of gastrodynia, a miserable complaint, which has been scarcely mentioned in medical books. For gastrodynia, medicine, with a single exception, is useless. The blue-pill, bitters, purgatives, antispasmodics, all, all are unavailing. Yet, fortunately for suffering humanity, fortunately for men of talents and genius, who are the greatest sufferers from it, there is a certain remedy; but, unluckily, being only palliative, it demands repetition. Had the efficacy of this remedy been sooner known to me, it would not have been my painful lot, as it now is, to write of racking paroxysms enduring for hours, or the principal part of a winter's night. The remedy is tincture of opium. Every one naturally flies to this drug in pain; yet its virtue has been unsuspected, once even by myself, in gastrodynia, in consequence of the smallness of the dose. In fact, the striking efficacy of opium, and the frequency of gastrodynia, amongst the higher ranks of life, are the causes why that medicine is so constantly and liberally taken by the statesmen, judges, lawyers, poets, authors, and actors, of the present day, whose names I conceal from delicacy. They undergo great mental exertion, and enjoy little exercise; meanwhile the stomach, that fruitful source of incalculable ills, disdains to be a passive spectator. I shall cite a single instance of the practice of taking opium, among the intellectual class; and for the truth of which I pledge my honour. A well-known living poet, sixteen years ago, occasionally walked into a druggist's shop, and swallowed there, with the most perfect indifference, one third of an ounce of Turkey opium, and two ounces of the tincture. His health appeared to be good: he sometimes took wine or spirits; but whether he now continues the use of opium, I am ignorant. A deceased and venerable judge, whilst sitting on the bench, has frequently been seen to drink the contents of a small phial, doubtless containing either the tincture of opium, or the black drop.

"When a patient suffers under a severe attack of gastrodynia—for, during the interval of ease opium is unnecessary,—he should immediately take one hundred drops of tincture in cold water, presuming he is a total stranger to that drug, and repeat half of the dose within three quarters of an hour, if the pain continue. The paroxysms are usually daily:—unfortunately, opium is not a preventive, and rarely effects a radical cure:—and, therefore, on their accession, however slight, the dose must be taken; for should it be omitted, the patient will, in a few hours, or next morning, suffer a most severe paroxysm; whereas if he take it, he will certainly remain well until the accustomed period of attack. The opium commonly induces constipation, which may be easily removed by daily draught of the compound infusion of senna, with quassia. The misfortune is, that a long continuance of the exhibition of opium requires a gradual augmentation of the dose; so that if a person begin with a drachm, and continue it daily or nightly for a year, he will then probably require five or six drachms for a dose. This is, however, unavoidable; and it is the same with stimulants of every class.

"It may be said, to take opium is a great evil—it is so; a most painful necessity: but it is a far greater evil to pass one half of life in excruciating pain, and the other half in miserable anticipation. It would be madness to reject a positive good for the possibility of a problematical and distant injury. The gastrodynic sufferer has a choice of evils: for him there is no middle path: he must either contentedly endure a pain which makes life a burthen and renders talents useless, or take opium;—for where is the man who, racked with pain in his stomach night and day, can perform his duties in society, and enjoy human life as it ought to be enjoyed? Again, where is the evil complained of? Excepting idiosyncrasies of constitution, opium apparently does good, and not harm; since it removes, within half an hour, a most agonizing pain, which would, without it, remain for hours, with the probability of a speedy return. It may be observed, certainly the patient is better during the influence of opium; but how does he feel hereafter? What is meant by hereafter?—the next day, or years? If the next day, the answer is most triumphant, inasmuch as the patient feels infinitely more lively and active than at any other period of his life:—if for years, the reply is not less triumphant, if we appeal to notorious facts, the only and best appeal in all doubtful cases, since almost all those who have indulged in the use of opium, probably from pain in the stomach, are now to be seen in the possession of good health and the enjoyment of old age. Many of them are either speaking in the senate, delivering judgment from the bench, exciting emotion on the stage, or producing works which rival those of antiquity.—This ought to be a conclusive answer to the individuals who imagine that opium injures the health and destroys the intellectual faculties.* The largeness of the dose is

"* The poet who took such enormous quantities of opium above sixteen years ago still lives, and continues to write admirable poems, one of which received the particular notice of Lord Byron."

sometimes objected to; but this is a most unphilosophical mode of argument, and founded upon ignorance of the principles of the animal economy. Medicines act on the system, according to the impression which they make on the part acted upon:—if therefore, one ounce of the tincture of opium produces the same effect as a single drachm once did, the one quantity is as harmless and influential as the other. At this moment a sufferer from gastrodynia is near me, who took last night eight drachms of tincture of opium with success and pleasure; and he assures me, that eleven months ago, when he commenced with it, sixty drops occasioned a more lasting and sensible effect.

"Where gastrodynia is the result of organic disease the effects of opium are temporary indeed, but most generally it is merely functional; and in such cases, incomparably more numerous, an adequate dose of opium speedily and completely dissipates the pain, invigorates the stomach, raises the spirits, and improves the general health: and when this pain returns, which it mostly does once in twenty-four hours, the repetition or augmentation of the dose again subdues it,—so that by such means gastrodynia is reduced to a most insignificant ailment; whereas if not so treated, it would probably terminate life in a few years by anxiety, sleeplessness, exhaustion, and pain. Far be it from my mind to advocate the cause of the unhappy sensualist, who, enduring no pain, takes opium merely for the pleasurable sensations which it excites; no,—I only advocate the cause of him, who, stretched on the rack of pain, swallows that drug to restore him to ease, to talents, to usefulness." 300.

In the above extract there is much that we can confirm by repeated experience—partly personal. To the therapeutic portion, *as regards opium*, we demur; but rather from theory than practice, as we candidly confess that we have not often given opium a fair trial. We are convinced, however, that Dr. Dawson has somewhat underrated the powers of other medicinal agents, at least in the less grievous forms of this distressing disease. We have found the volatile alkali, in conjunction with magnesia, mint water, and occasionally, the tincture of henbane, very useful, especially when a rigid animal food diet, and a moderate allowance of good wine, or brandy and water, were carefully adhered to. The sulphate of quinine we have recently found an excellent medicine in gastrodynia.

We must now part from our author, not without being impressed with great respect for his talents, erudition, and professional judgment. The work itself, though rather too concise, contains, we will venture to affirm, more correct pathology and sound practice than any systematic work of the same size in the English language.

